# Deaths Associated with Pandemic (H1N1) 2009 among Children, Japan, 2009–2010

**DOI:** 10.3201/eid1711.110649

**Published:** 2011-11

**Authors:** Akihisa Okumura, Satoshi Nakagawa, Hisashi Kawashima, Takashi Muguruma, Osamu Saito, Jun-ichi Fujimoto, Chiaki Toida, Shuji Kuga, Toshihiro Imamura, Toshiaki Shimizu, Naomi Kondo, Tsuneo Morishima

**Affiliations:** Author affiliations: Juntendo University Faculty of Medicine, Tokyo, Japan (A. Okumura, T. Shimizu);; National Center for Child Health and Development, Tokyo (S. Nakagawa, T. Muguruma, O. Saito, J. Fujimoto, C. Toida, S. Kuga, T. Imamura);; Tokyo Medical University, Tokyo (H. Kawashima);; Gifu University Graduate School of Medicine, Gifu, Japan (N. Kondo);; Okayama University Graduate School of Medicine, Dentistry and Pharmaceutical Sciences, Okayama, Japan (T. Morishima)

**Keywords:** Pediatric death, pandemic (H1N1) 2009, cause of death, influenza, preexisting conditions, children, viruses, unexpected cardiopulmonary arrest, encephalopathy, pneumonia, Japan, research

## Medscape CME

Medscape, LLC is pleased to provide online continuing medical education (CME) for this journal article, allowing clinicians the opportunity to earn CME credit.

This activity has been planned and implemented in accordance with the Essential Areas and policies of the Accreditation Council for Continuing Medical Education through the joint sponsorship of Medscape, LLC and Emerging Infectious Diseases. Medscape, LLC is accredited by the ACCME to provide continuing medical education for physicians.

Medscape, LLC designates this Journal-based CME activity for a maximum of 1 *AMA PRA Category 1 Credit(s)*^TM^. Physicians should claim only the credit commensurate with the extent of their participation in the activity.

All other clinicians completing this activity will be issued a certificate of participation. To participate in this journal CME activity: (1) review the learning objectives and author disclosures; (2) study the education content; (3) take the post-test with a 70% minimum passing score and complete the evaluation at www.medscape.org/journal/eid; (4) view/print certificate.

**Release date: October 21, 2011; Expiration date: October 21, 2012**

## Learning Objectives

Upon completion of this activity, participants will be able to:

- Distinguish the most common presenting symptom in fatal cases of pandemic (H1N1) 2009 infection among children
- Assess the most common causes of death among children with pandemic (H1N1) 2009 infection
- Analyze the causes of death in fatal cases of pandemic (H1N1) 2009 infection among children.

## Editor

**Caran Wilbanks,** Technical Writer/Editor, *Emerging Infectious Diseases. Disclosure: Caran Wilbanks has disclosed the following relevant financial relationships: partner is employed by McKesson Corporation*.

## Medscape CME AUTHOR

**Charles P. Vega, MD**, Associate Professor; Residency Director, Department of Family Medicine, University of California, Irvine. *Disclosure: Charles P. Vega, MD, has disclosed no relevant financial relationships.*

## AUTHORS

*Disclosures:* ***Akihisa Okumura, MD, PhD; Satoshi Nakagawa MD, PhD; Hisashi Kawashima, MD, PhD; Takashi Muguruma, MD, PhD; Osamu Saito, MD; Jun-ichi Fujimoto, MD; Chiaki Toida, MD; Shuji Kuga, MD; Toshihiro Imamura, MD; Toshiaki Shimizu, MD, PhD; Naomi Kondo, MD, PhD;*** *and* ***Tsuneo Morishima, MD, PhD,*** *have disclosed no relevant financial relationships.*
