# Supplementary material for: Deaths Associated with Pandemic (H1N1) 2009 among Children, Japan, 2009–2010
Source: Emerg Infect Dis. 2011 Nov;17(11):1993–2000. doi: 10.3201/eid1711.110649 (PMC3311116; doi:10.3201/eid1711.110649)
Supplement: Related texts

# Research article: Deaths Associated with Pandemic (H1N1) 2009 among Children, Japan, 2009–2010

**DOI:** 10.3201/eid1711.110649

**Authors:** Akihisa Okumura, Satoshi Nakagawa, Hisashi Kawashima, Takashi Muguruma, Osamu Saito, Jun-ichi Fujimoto, Chiaki Toida, Shuji Kuga, Toshihiro Imamura, Toshiaki Shimizu, Naomi Kondo, Tsuneo Morishima

**Affiliations:** Author affiliations: Juntendo University Faculty of Medicine, Tokyo, Japan (A. Okumura, T. Shimizu);; National Center for Child Health and Development, Tokyo (S. Nakagawa, T. Muguruma, O. Saito, J. Fujimoto, C. Toida, S. Kuga, T. Imamura);; Tokyo Medical University, Tokyo (H. Kawashima);; Gifu University Graduate School of Medicine, Gifu, Japan (N. Kondo);; Okayama University Graduate School of Medicine, Dentistry and Pharmaceutical Sciences, Okayama, Japan (T. Morishima)

## Abstract

To clarify the cause of deaths associated with pandemic (H1N1) 2009 among children in Japan, we retrospectively studied 41 patients <20 years of age who had died of pandemic (H1N1) 2009 through March 31, 2010. Data were collected through interviews with attending physicians and chart reviews. Median age of patients was 59 months; one third had a preexisting condition. Cause of death was categorized as unexpected cardiopulmonary arrest for 15 patients, encephalopathy for 15, and respiratory failure for 6. Preexisting respiratory or neurologic disorders were more frequent in patients with respiratory failure and less frequent in patients with unexpected cardiopulmonary arrest. The leading causes of death among children with pandemic (H1N1) 2009 in Japan were encephalopathy and unexpected cardiopulmonary arrest. Deaths associated with respiratory failure were infrequent and occurred primarily among children with preexisting conditions. Vaccine use and public education are necessary for reducing influenza-associated illness and death.

A novel reassortant strain of influenza A (H1N1) virus containing swine, avian, and human elements (*1*) emerged in Mexico in March 2009. The virus initially spread within North America, causing severe respiratory illnesses in Mexico (*2*) and the United States (*3**,**4*), and then began to spread rapidly worldwide. On June 11, 2009, the World Health Organization confirmed an influenza pandemic.

In Japan, the first case of pandemic (H1N1) 2009 was confirmed on May 16, 2009. The first outbreak occurred in western Japan, where the number of cases increased then decreased quickly. The second outbreak started in early June and quickly spread to all parts of Japan. The first death associated with pandemic (H1N1) 2009 in Japan was confirmed August 15, and the first death of a child occurred September 17. As of March 31, 2010, the Ministry of Health, Labour, and Welfare (MHLW) reported on its website (www.mhlw.go.jp/kinkyu/kenkou/influenza/houdou.html) that 198 patients in Japan with pandemic (H1N1) 2009 had died, of whom 41 were children <20 years of age.

Several authors have reported that respiratory diseases associated with pandemic (H1N1) 2009, including viral pneumonia and acute lung injury, that required intensive care occurred most often in children (*5**–**16*). In Japan, hospitalizations of children because of severe pneumonia or other respiratory complications increased (*17*). Concerns were raised regarding deaths among children from acute encephalopathy in association with pandemic (H1N1) 2009 because acute encephalopathy has been associated with death from seasonal influenza in Japan (*18**,**19*). Neurologic complications associated with pandemic (H1N1) 2009, including acute encephalopathy, altered mental status, and status epilepticus, also have been reported from other countries (*20**–**23*).

Accurate data on the causes of death associated with pandemic (H1N1) 2009 among children are necessary for making a counterplan against future pandemic influenza. We investigated detailed clinical data collected by MHLW for children whose deaths were associated with pandemic (H1N1) 2009. We focused on the direct cause of death and clinical differences by cause of death.

## Materials and Methods

After the first patient was identified in May 2009, all medical professionals were required to report deaths associated with pandemic (H1N1) 2009 to MHLW. Press releases on patient deaths were provided on MHLW’s website. As of March 31, 2010, a total of 41 patients <20 years of age were listed.

Infection with pandemic (H1N1) 2009 virus was confirmed with nasal swab specimens or aspirates from the nose, throat, or tracheal tube by using real-time reverse transcription PCR (RT-PCR) at local public health laboratories or the National Institute of Infectious Diseases in Japan, according to the institute’s recommended protocol. Samples for RT-PCR could not be obtained for 3 patients; however, rapid antigen tests were positive for influenza A for all 41 patients. Because influenza A viruses other than pandemic (H1N1) 2009 virus were rarely isolated in Japan during the study, these 3 patients were included in our analysis.

Two research groups collaborated to collect detailed data on deaths associated with pandemic (H1N1) 2009 among children. The collaborative study group comprised 3 chief members (A.O., S.N., and H.K.) and 6 assistant members (T.M., O.S., J.F., C.T., S.K., and T.I.). During February–June 2010, members of the collaborative study group contacted the attending physician of each child who died and visited the hospital to obtain detailed information. We abstracted data from medical records by using a structured report form and obtained demographic, clinical, laboratory, and radiologic data from interviews with attending physicians and chart reviews. Onset of influenza was considered the time at which a temperature >38°C was first recorded. The chief members of the study group reevaluated chest radiographs; computed tomography (CT) scan of the head, chest, and abdomen; and magnetic resonance images of the head, including those obtained at autopsy.

Cause of death (Table 1) for each patient was categorized after the 3 chief members reviewed the detailed clinical course and laboratory and radiologic data. At first, each chief member independently presumed the cause of death for each patient. When they agreed on the presumed cause of death, it was adopted as a cause of death. When the chief members disagreed, they reached a consensus on the cause of death after discussion.

**Table 1:** Causes of death for 41 patients <20 years of age with pandemic (H1N1) 2009, Japan, May 2009–March 2010

Cause	Definition
Unexpected cardiopulmonary arrest	Cardiopulmonary arrest without clear findings of respiratory failure, cardiomyopathy, or encephalopathy
Respiratory failure	Desaturation, need for oxygen supplementation or mechanical ventilation or both, associated with radiologic findings of pneumonia or acute lung injury
Myocarditis	Markedly reduced cardiac output, severe and refractory arrhythmia, or severe circulatory collapse
Viral sepsis	Refractory hypotension and rapidly progressing multiorgan failure associated with at least 2 of the following: tachypnea; leukopenia <4000 cells/μL or leukocytosis >12,000 cells/μL; tachycardia; body temperature >38.0°C or <36.0°C; cold extremities; and increased capillary refill time
Encephalopathy	At least 1 of the following: altered mental state without profound respiratory and cardiac failure or neuroimaging findings consistent with encephalopathy such as marked brain edema, focal lesions, and blurred gray-white matter junction
Incidental	Other findings that are not directly attributable to influenza infection

Because the study was considered to be a public health activity entailing surveillance of deceased persons, approval from an ethics committee or institutional review boards at participating hospitals and informed consent were not required. Anonymous data were collected retrospectively and were kept confidential.

Statistical analyses were performed to identify differences among patients by cause of death. Because the number of patients who died of myocarditis and viral sepsis was small, these cases were excluded from statistical analyses. We also excluded 1 patient who died of presumed incidental intracranial hemorrhage. The Kruskal-Wallis test was used to compare numerical variables. When a p value <0.05 was obtained by Kruskal-Wallis test, post hoc testing was performed by using the Tukey test. We compared categorical variables by using the χ^2^ test. When the χ^2^ test gave a p value <0.05, adjustment residual analysis was performed. An absolute value of the adjustment residual >2 was considered significant.

## Results

### Study Population

Deaths included in the study were distributed almost evenly throughout Japan. The timing of infection with pandemic (H1N1) 2009 virus was concentrated primarily during October 2009–January 2010 (Figure 1).

**Figure 1:** Timing of onset of pandemic (H1N1) 2009 in children and patient age, Japan, May 2009–March 2010. A) Date of illness onset for children >15 years of age compared with those <15 years of age. B) No. patients at each age at time of illness onset.

Median age of children was 59 months (range 7–206 months); 20 (49%) patients were 0–4 years of age and 12 (29%) were 5–9 years of age (Figure 1). Twenty-five (61%) patients were boys. Fourteen (34%) patients had >1 preexisting conditions. Respiratory disorders (at least 1 of asthma, chronic lung disease, or a disorder necessitating tracheostomy) were present in 9 patients, none of whom were receiving systemic corticosteroids. Neurologic disorders (at least 1 of cerebral palsy, mental retardation, epilepsy, or neuromuscular disease) were present in 11 patients, 9 of whom had >2 neurologic disorders, and 7 had concurrent respiratory disorders. No patients had endocrine or immunologic disorders or obesity. History of febrile seizures was noted for 6 (15%) patients. One patient had been vaccinated against pandemic (H1N1) 2009 virus and another 2 against seasonal influenza virus. The other 38 had not been vaccinated against pandemic (H1N1) 2009 virus or seasonal influenza virus. Close contact with a person who had influenza within a few days before symptom onset was reported for 15 (44%) of 34 patients for whom this information was available.

Information about clinical signs and symptoms of infection with pandemic (H1N1) 2009 virus was available for all but 1 patient. Clinical signs included temperature >38°C (40 [100%] patients), cough (20 [50%]), rhinorrhea (12 [30%]), tachypnea (10 [25%]), dyspnea (12 [30%]), and wheezing (6 [15%]). Vomiting was observed in 8 (20%) patients; diarrhea (3 patients), tachycardia (3), headache (1), and myalgia (2) were rare.

Influenza was diagnosed by rapid antigen test within 2 days after onset of fever for 39 (95%) patients. Before the life-threatening event, 19 (46%) patients received oseltamivir and 5 (12%) received zanamivir. These antiviral drugs were prescribed soon after diagnosis of influenza by rapid antigen test. Acetaminophen was administered to 13 (39%) of 33 patients for whom this information was available.

For 34 (83%) children, a life-threatening event occurred within 2 days after influenza onset (Figure 2). Twenty-nine (71%) children died within 4 days after influenza onset (Figure 2). Patient age, interval between onset of fever and life-threatening event, or interval between onset of fever and death did not differ by presence or absence of preexisting conditions. Death was confirmed in an emergency department for 14 patients, intensive care unit for 13, inpatient ward for 12, outside of a hospital for 1, and outpatient clinic for 1.

**Figure 2:** Days from onset of pandemic (H1N1) 2009 illness to A) life-threatening event or B) death among patients <20 years of age, Japan, May 2009–March 2010.

Blood culture test results were positive for only 1 of 21 patients who had at least 1 blood culture; this patient had had pneumonia associated with methicillin-resistant *Staphylococcus* *aureus* (MRSA) before infection with pandemic (H1N1) 2009 virus. Bacterial cultures from respiratory tract samples were positive for 2 of 16 patients (1 with MRSA and 1 with *Streptococcus pneumoniae* infection). Information about pathologic findings was not available for any of the 6 patients for whom postmortem examinations were conducted.

### Causes of Death

Cause of death was categorized as unexpected cardiopulmonary arrest (CPA) for 15 patients, encephalopathy for 15, respiratory failure for 6, myocarditis for 2, viral sepsis for 2, and incidental for 1. Median age of patients who died of unexpected CPA was 43 months. Only 1 of these patients had a preexisting condition. For 13 patients, unexpected CPA occurred outside the hospital; most patients were presumed to have been found several hours after CPA. Two patients experienced unexpected CPA in the hospital, 1 in the outpatient clinic and 1 during hospitalization. Chest radiographs and CT scans of the head and chest were unremarkable for all children examined.

Encephalopathy was considered the cause of death for 15 patients (median age 62 months). Five of these patients had a preexisting condition, and 3 had preexisting neurologic disorders. All 15 patients had altered mental state or convulsions or both and marked brain edema according to head CT scan or magnetic resonance images or both, which suggests increased intracranial pressure. Nine patients also had low-density areas in the bilateral thalami or brainstem or both. Most patients had clinical brain death within several hours after onset of encephalopathy in association with multiple organ failure. For some patients, mild infiltration was seen on chest radiograph, but pulmonary involvement was not likely the cause of death.

Six patients were judged to have died of respiratory failure; their median age was 78 months. Five of these patients had preexisting neurologic conditions and had radiologic findings consistent with severe pneumonia. Two had been hospitalized because of pneumonia attributable to other pathogens (MRSA for 1 and undetermined for the other) before infection with pandemic (H1N1) 2009 virus; their respiratory state markedly worsened after infection. Nosocomial transmission was strongly suspected, and influenza was diagnosed for both patients on the day after fever onset. One previously healthy patient had severe and rapidly progressive dyspnea and hypoxemia. Chest CT scan performed at autopsy indicated severe infiltration in the entire lungs, corresponding to acute lung injury.

Two patients died of myocarditis; both were >12 years of age and previously healthy. One had unexpected circulatory collapse in a local pediatric clinic; the other was found lying on the floor at home without preceding respiratory or neurologic symptoms. At admission, both patients had markedly elevated creatine kinase (>9,000 IU/L) and markedly reduced cardiac output on cardiac ultrasonography; chest radiographs were unremarkable. In the clinic patient, intensive resuscitation, including intra-aortic balloon pumping and continuous hemodiafiltration, was performed but was ineffective.

Viral sepsis resulting from pandemic (H1N1) 2009 virus developed in 2 patients; 1 was severely disabled. Tachypnea, cold extremities, and lethargy were noted for both patients at the local pediatric clinic; shock was diagnosed, and they were immediately transferred to tertiary emergency hospitals. Both had rapidly progressive multiple organ failure with refractory hypotension. For both patients, chest radiographs were unremarkable.

Cause of death was presumed to be incidental to pandemic (H1N1) 2009 virus infection for 1 patient. This patient was hospitalized because of intracranial hemorrhage, which neuroimaging suggested resulted from rupture of an arteriovenous malformation. On day 12 of illness, infection with pandemic (H1N1) 2009 virus was confirmed by RT-PCR.

### Comparisons by Cause of Death

We compared demographic and laboratory data of 36 patients by cause of death (Table 2). Patients who died of myocarditis, viral sepsis, or incidental intracranial hemorrhage were excluded. Patients with unexpected CPA were younger than other patients, although these differences were not significant (p = 0.053). Respiratory or neurologic disorders occurred significantly more often in patients with respiratory failure and significantly less often in patients with unexpected CPA. The interval between influenza onset and life-threatening event did not differ by cause of death. Most life-threatening events occurred on the day of or 1 day after influenza onset. Although the percentage of clinical signs and symptoms did not differ by cause of death, tachypnea/dyspnea or wheezing were frequent in patients with respiratory failure. Drugs taken before a life-threatening event did not differ by cause of death. Leukocyte and platelet counts did not differ by cause of death. Alanine transaminase and creatine kinase levels were significantly higher in patients with unexpected CPA than in those with respiratory failure. Blood urea nitrogen concentration was significantly higher in patients with encephalopathy than in those with unexpected CPA or respiratory failure. Levels of aspartate aminotransferase, lactate dehydrogenase, and creatinine did not differ by cause of death.

**Table 2:** Comparisons by cause of death among patients <20 years of age, Japan, May 2009–March 2010*

Characteristic	Unexpected CPA, n = 15†	Encephalopathy, n = 15†	Respiratory failure, n = 6†	p value
Median age, mo (range)	43 (7–164)	62 (17–200)	78 (45–206)	0.053
Male sex, no. (%) patients	9 (60)	10 (67)	2 (33)	NS
Preexisting condition, no. (%) patients				
Any	1 (7)‡	5 (33)	5 (83)§	<0.05
Respiratory disorders	0‡	3 (20)	4 (67)§	<0.05
Neurologic disorders	1 (7)‡	3 (20)	5 (83)§	<0.05
Previous history of febrile seizures	2 (13)	3 (20)	0	NS
Days from influenza onset to life-threatening event (range)	1 (0–9)	1 (0–2)	1 (0–9)	NS
Days from influenza onset to death (range)	1 (0–9)	3 (0–45)	1.5 (1–11)	<0.05¶
Clinical signs, no. (%) patients				
Cough	5 (36), n = 14	11 (73)	3 (50)	NS
Rhinorrhea	4 (29), n = 14	7 (47)	1 (17)	NS
Tachypnea or dyspnea	1 (7), n = 14	4 (27)	3 (50)	NS
Wheezing	1 (7), n = 14	1 (7)	2 (33)	NS
Vomiting or diarrhea	2 (14), n = 14	5 (33)	0	NS
Drugs received before life-threatening event, no. (%) patients				
Oseltamivir	7 (47)	6 (40)	5 (83)	NS
Zanamivir	1 (7)	2 (13)	2 (33)	NS
Acetaminophen	3 (30), n = 10	7 (50), n = 14	2 (40), n = 5	NS
Leukocyte count, cells/μL, median (range)	6,600 (4,200–11,100), n = 11	9,350 (3,100–28,730), n = 14	10,500 (8,650–101,200), n = 5	NS
Platelet count, × 10^4^ cells/μL, median (range)	20.5 (11.4–45.8), n = 11	15.1 (6.2–32.2)	11.5 (8.4–49.0), n = 5	NS
Aspartate aminotransferase, IU/L, median (range)	248 (55–1,981), n = 12	233 (18–1,760)	52 (34–73), n = 4	0.060
Alanine transaminase, IU/L, median (range)	157 (32–845), n = 12	70 (9–1,058)	26 (16–57), n = 4	<0.05¶
Lactate dehydrogenase, IU/L, median (range)	704 (215–4,801), n = 12	899 (160–3,610), n = 14	535 (222–1,022), n = 4	NS
Creatine kinase, IU/L, median (range)	302 (136–10,612), n = 12	190 (63–1,026), n = 14	64 (16–211), n = 4	<0.01#
Blood urea nitrogen, mg/dL, median (range)	11.2 (7.0–31.0), n = 12	22.5 (11.7–40.0), n = 14	10.0 (4.0–15.0), n = 4	<0.01**
Creatinine, mg/dL, median (range)	0.65 (0.17–1.40), n = 12	1.01 (0.62–1.39)	0.25 (0.08–1.00), n = 4	<0.05**

*CPA, cardiopulmonary arrest; NS, not significant. †n is for all values unless indicated otherwise. ‡Percentage significantly lower than for the other groups. §Percentage significantly higher than for the other groups. ¶p<0.05 unexpected CPA vs. encephalopathy. #p<0.05 unexpected CPA vs. respiratory failure. **p<0.01 unexpected CPA vs. encephalopathy; p<0.05 respiratory failure vs. encephalopathy.

## Discussion

We investigated the causes of death associated with pandemic (H1N1) 2009 among children in Japan. Most cases were in young, previously healthy children who died after a brief fulminant illness. Unexpected CPA and acute encephalopathy were the leading causes of death. Children who died of respiratory failure often had preexisting conditions, whereas unexpected CPA occurred among younger children without preexisting conditions.

Our finding that encephalopathy was a leading cause of death associated with pandemic (H1N1) 2009 among children in Japan differs from reports from other countries that few children have died of neurologic complications (*5**,**24*). Children with acute encephalopathy or encephalitis associated with pandemic (H1N1) 2009 have been reported outside Japan (*20**–**23*), but most survived with no or mild neurologic sequelae. Most children with acute encephalopathy, such as acute necrotizing encephalopathy (*25*) and acute encephalopathy with biphasic seizures and late reduced diffusion (*26*), were of Japanese or east Asian descent. Children in Japan are presumed to have an underlying genetic predisposition for development of acute encephalopathy (*26*). The median age of children who died of encephalopathy (62 months) was older than that of patients with encephalopathy associated with seasonal influenza (median 2–3 years) (*19**,**27*). This difference in age may be related to the age of infected patients; in Japan, more patients 5–9 years or 10–14 years of age were infected with pandemic (H1N1) 2009 than were those 0–4 years (*28*). A fulminant clinical course and marked brain edema were characteristic and common in the encephalopathy patients in our study, irrespective of age, presence or absence of preexisting conditions, and neuroradiologic findings.

Unexpected CPA was another leading cause of death associated with pandemic (H1N1) 2009 among children in Japan. Most cases of unexpected CPA occurred in previously healthy children <5 years of age. The elevated alanine transaminase and creatine kinase levels in these children could be attributable to postmortem changes. The direct cause of unexpected CPA is difficult to determine. One possible explanation is severe brain damage resulting in CPA; however, none of the patients in our study had obvious neurologic signs or symptoms until CPA, nor did they have any evidence of brain herniation. Abrupt onset of CPA suggests a cardiogenic origin such as fatal arrhythmia from undetected myocarditis (*29**,**30*). Myocarditis associated with pandemic (H1N1) 2009 has been reported (*31**,**32*). Gdynia et al. reported an unexpected death of a young adult caused by pandemic (H1N1) 2009–associated myocarditis (*31*). The clinical course in this patient was characterized by sudden collapse at home followed by fatal arrhythmia. Viral sepsis may also be related to unexpected CPA. Clinical signs of viral sepsis are nonspecific and may be missed. Considering that most cases of unexpected CPA occurred outside the hospital, rapid progression of viral sepsis may have occurred. Unexpected CPA has also been reported in some case series (*5**–**7**,**24**,**33*). Cardiac arrest outside the hospital was observed for 67 of 270 children who died in the United States (*24*). In a report from England, 16 of 70 children who died were in CPA when seen in an emergency department (*7*). Detailed postmortem examinations are necessary to clarify the mechanism of unexpected CPA.

Respiratory failure was an uncommon cause of death among children in Japan. In other countries, diffuse viral pneumonia or pneumonitis with severe hypoxemia were strongly associated with intensive care unit admission associated with pandemic (H1N1) 2009 (*8**–**13*). Several reports on cases of pandemic (H1N1) 2009 in children also showed that respiratory distress is most common among hospitalized children (*5**–**7**,**14**–**16**,**24**,**34**,**35*). In a study of children in Argentina, refractory hypoxemia caused 62% of all deaths (*14*). A report from England described predominantly respiratory symptoms when care was sought in 53 of 70 children who died (*7*). Most children who died of respiratory failure in Japan had preexisting neurologic or respiratory disorders or both; this finding is similar to reports from other countries (*5**–**7**,**10**,**12**,**14**–**16**,**24**,**34*). Children with preexisting neurologic or respiratory disorders are at increased risk for severe illness or death with influenza, and influenza vaccination should be a priority for these children.

The infrequency of preexisting conditions appears to be a feature of deaths associated with pandemic (H1N1) 2009 among children in Japan. Only one third of the participants in our study had >1 preexisting conditions. In contrast, research in Argentina showed that 9 of 13 patients who died of pandemic (H1N1) 2009 had a preexisting condition, including neurologic disorders and chronic lung disease (*14*). In England, preexisting severe or incapacitating systemic diseases were recognized in all deaths in children <5 years of age and in most of those 5–14 years of age (*15*). According to the US Centers for Disease Control and Prevention, 205 of 301 children in the United States whose deaths occurred in association with pandemic (H1N1) 2009 had high-risk medical conditions as defined by the Advisory Committee on Immunization Practices (*24*).

The strength of our study is use of the detailed and precise information obtained during interviews with attending clinicians. Clinical course and demographic data were accurate and detailed, and laboratory data, chest radiographs, and other radiologic data were directly assessed by the study team. Data were standardized by use of a structured report form. In addition, cause of death was determined on the basis of the consensus of the chief study members rather than by the attending clinicians. We thus consider that the data from our study are objective.

Nevertheless, our study has some limitations. First, in some cases, infection might not have been confirmed by PCR; thus, the number of deaths associated with pandemic (H1N1) 2009 among children might have been underestimated. Second, complete data on the number of all children infected with pandemic (H1N1) 2009 virus were not available. In Japan, data on the number of patients with influenza-like symptoms are collected from ≈3,000 sentinel pediatric physicians and 2,000 sentinel internal medicine physicians participating in the surveillance system. Because an accurate number of all infected children could not be obtained, the case-fatality rate could not be determined.

Several authors have suggested that neuraminidase inhibitors will be effective for preventing severe illness in patients with pandemic (H1N1) 2009 virus infection (*36**,**37*), and the usefulness of early treatment with neuraminidase inhibitors has been emphasized. However, neuraminidase inhibitors did not appear to be effective in our patients, even though the drugs had been used without delay, which indicates the difficulty of improving the outcome for children with the most severe illness. Prevention and control of influenza with vaccine use and public education is necessary for reducing illness and deaths associated with influenza not only in high-risk children but also in previously healthy ones.

## References

[1] Novel Swine-Origin Influenza A (H1N1) Virus Investigation Team; Dawood FS, Jain S, Finelli L, Shaw MW, Lindstrom S, et al. Emergence of a novel swine-origin influenza A (H1N1) virus in humans [erratum in: N Engl J Med. 2009;361:102]. N Engl J Med. 2009;360:2605–15. 10.1056/NEJMoa090381019423869

[2] Echevarría-Zuno S, Mejía-Aranguré JM, Mar-Obeso AJ, Grajales-Muñiz C, Robles-Pérez E, González-León M, Infection and death from influenza A H1N1 virus in Mexico: a retrospective analysis. Lancet. 2009;374:2072–9. 10.1016/S0140-6736(09)61638-X19913290

[3] Jamieson DJ, Honein MA, Rasmussen SA, Williams JL, Swerdlow DL, Biggerstaff MS, H1N1 2009 influenza virus infection during pregnancy in the USA. Lancet. 2009;374:451–8. 10.1016/S0140-6736(09)61304-019643469

[4] Centers for Disease Control and Prevention. Preliminary information important for understanding the evolving situation: pandemic (H1N1) 2009 briefing note 4, July 24, 2009 [cited 2009 Dec 10]. http://www.cdc.gov/H1N1flu/updates/us

[5] Yung M, Slater A, Festa M, Williams G, Erickson S, Pettila V, Pandemic H1N1 in children requiring intensive care in Australia and New Zealand during winter 2009. Pediatrics. 2011;127:e156–63. 10.1542/peds.2010-080121172991

[6] Louie JK, Gavali S, Acosta M, Samuel MC, Winter K, Jean C, Children hospitalized with 2009 novel influenza A(H1N1) in California. Arch Pediatr Adolesc Med. 2010;164:1023–31. 10.1001/archpediatrics.2010.20321041595

[7] Sachedina N, Donaldson LJ. Paediatric mortality related to pandemic influenza A H1N1 infection in England: an observational population-based study. Lancet. 2010;376:1846–52. 10.1016/S0140-6736(10)61195-621030071

[8] Nguyen-Van-Tam JS, Openshaw PJ, Hashim A, Gadd EM, Lim WS, Semple MG, Risk factors for hospitalisation and poor outcome with pandemic A/H1N1 influenza: United Kingdom first wave (May–September 2009). Thorax. 2010;65:645–51. 10.1136/thx.2010.13521020627925PMC2921287

[9] ANZIC Influenza Investigators; Webb SA, Pettilä V, Seppelt I, Bellomo R, Bailey M, et al. Critical care services and 2009 H1N1 influenza in Australia and New Zealand. N Engl J Med. 2009;361:1925–34. 10.1056/NEJMoa090848119815860

[10] Kumar A, Zarychanski R, Pinto R, Cook DJ, Marshall J, Lacroix J, Critically ill patients with 2009 influenza A(H1N1) infection in Canada. JAMA. 2009;302:1872–9. 10.1001/jama.2009.149619822627

[11] Perez-Padilla R, de la Rosa-Zamboni D, Ponce de Leon S, Hernandez M, Quiñones-Falconi F, Bautista E, Pneumonia and respiratory failure from swine-origin influenza A (H1N1) in Mexico. N Engl J Med. 2009;361:680–9. 10.1056/NEJMoa090425219564631

[12] Louie JK, Acosta M, Winter K, Jean C, Gavali S, Schechter R, Factors associated with death or hospitalization due to pandemic 2009 influenza A(H1N1) infection in California. JAMA. 2009;302:1896–902. 10.1001/jama.2009.158319887665

[13] Writing Committee of the WHO Consultation on Clinical Aspects of Pandemic(H1N1) 2009 Influenza; Bautista E, Chotpitayasunondh T, Gao Z, Harper SA, Shaw M, et al. Clinical aspects of pandemic 2009 influenza A (H1N1) virus infection. N Engl J Med. 2010;362:1708–19. 10.1056/NEJMra100044920445182

[14] Libster R, Bugna J, Coviello S, Hijano DR, Dunaiewsky M, Reynoso N, Pediatric hospitalizations associated with 2009 pandemic influenza A (H1N1) in Argentina. N Engl J Med. 2010;362:45–55. 10.1056/NEJMoa090767320032320

[15] Donaldson LJ, Rutter PD, Ellis BM, Greaves FE, Mytton OT, Pebody RG, Mortality from pandemic A/H1N1 2009 influenza in England: public health surveillance study. BMJ. 2009;339:b5213. 10.1136/bmj.b521320007665PMC2791802

[16] Jouvet P, Hutchison J, Pinto R, Menon K, Rodin R, Choong K, Critical illness in children with influenza A/pH1N1 2009 infection in Canada. Pediatr Crit Care Med. 2010;11:603–9. 10.1097/PCC.0b013e3181d9c80b20308929

[17] The Japan Pediatric Society The recent trend of pandemic influenza A (H1N1) 2009 among children [in Japanese] [cited 2011 Feb 28]. http://www.jpeds.or.jp/influenza/influenza_091119.pdf

[18] Kasai T, Togashi T, Morishima T. Encephalopathy associated with influenza epidemics. Lancet. 2000;355:1558–9. 10.1016/S0140-6736(05)74614-610801205

[19] Morishima T, Togashi T, Yokota S, Okuno Y, Miyazaki C, Tashiro M, Encephalitis and encephalopathy associated with an influenza epidemic in Japan. Clin Infect Dis. 2002;35:512–7. 10.1086/34140712173123

[20] Ekstrand JJ, Herbener A, Rawlings J, Turney B, Ampofo K, Korgenski EK, Heightened neurologic complications in children with pandemic H1N1 influenza. Ann Neurol. 2010;68:762–6. 10.1002/ana.2218420865762

[21] Centers for Disease Control and Prevention. Neurologic complications associated with novel influenza A (H1N1) virus infection in children—Dallas, Texas, May 2009. MMWR Morb Mortal Wkly Rep. 2009;58:773–8.19629027

[22] Rellosa N, Bloch KC, Shane AL, Debiasi RL. Neurologic manifestations of pediatric novel H1N1 influenza infection. Pediatr Infect Dis J. 2011;30:165–7. 10.1097/INF.0b013e3181f2de6f20811314

[23] Baltagi SA, Shoykhet M, Felmet K, Kochanek PM, Bell MJ. Neurological sequelae of 2009 influenza A (H1N1) in children: a case series observed during a pandemic. Pediatr Crit Care Med. 2010;11:179–84. 10.1097/PCC.0b013e3181cf465220081552

[24] Cox CM, Blanton L, Dhara R, Brammer L, Finelli L. 2009 Pandemic influenza A (H1N1) deaths among children—United States, 2009–2010. Clin Infect Dis. 2011;52(Suppl 1):S69–74. 10.1093/cid/ciq01121342902

[25] Mizuguchi M. Acute necrotizing encephalopathy of childhood: a novel form of acute encephalopathy prevalent in Japan and Taiwan. Brain Dev. 1997;19:81–92. 10.1016/S0387-7604(96)00063-09105653

[26] Traul DE, Traul CS, Matsumoto J, Goodkin HP. Acute encephalopathy with biphasic seizures and late restricted diffusion on MRI in a Japanese child living in the USA. Dev Med Child Neurol. 2008;50:717–9. 10.1111/j.1469-8749.2008.03080.x18754924

[27] Togashi T, Matsuzono Y, Narita M. Epidemiology of influenza-associated encephalitis–encephalopathy in Hokkaido, the northernmost island of Japan. Pediatr Int. 2000;42:192–6. 10.1046/j.1442-200x.2000.01202.x10804741

[28] Kamigaki T, Oshitani H. Epidemiological characteristics and low case fatality rate of pandemic (H1N1) 2009 in Japan. PLoS Curr. 2009;1:RRN1139. 10.1371/currents.RRN113920043033PMC2797432

[29] Nolte KB, Alakija P, Oty G, Shaw MW, Subbarao K, Guarner J, Influenza A virus infection complicated by fatal myocarditis. Am J Forensic Med Pathol. 2000;21:375–9. 10.1097/00000433-200012000-0001611111801

[30] Lajoie J, Sagy M, Gonzalez R. Reye’s syndrome associated with acute myocarditis and fatal circulatory failure. Pediatr Emerg Care. 1991;7:226–8. 10.1097/00006565-199108000-000081758777

[31] Gdynia G, Schnitzler P, Brunner E, Kandolf R, Bläker H, Daum E, Sudden death of an immunocompetent young adult caused by novel (swine origin) influenza A/H1N1–associated myocarditis. Virchows Arch. 2011;458:371–6. 10.1007/s00428-010-1035-021234762

[32] Kumar K, Guirgis M, Zieroth S, Lo E, Menkis AH, Arora RC, Influenza myocarditis and myositis: case presentation and review of the literature. Can J Cardiol. 2011;27:514–22. 10.1016/j.cjca.2011.03.00521652168

[33] Centers for Disease Control and Prevention. Surveillance for pediatric deaths associated with 2009 pandemic influenza A (H1N1) virus infection—United States, April–August 2009. MMWR Morb Mortal Wkly Rep. 2009;58:941–7.19730406

[34] Lister P, Reynolds F, Parslow R, Chan A, Cooper M, Plunkett A, Swine-origin influenza virus H1N1, seasonal influenza virus, and critical illness in children. Lancet. 2009;374:605–7. 10.1016/S0140-6736(09)61512-919700000

[35] Larcombe PJ, Moloney SE, Schmidt PA. Pandemic (H1N1) 2009: a clinical spectrum in the general paediatric population. Arch Dis Child. 2011;96:96–8. 10.1136/adc.2009.17685919906639

[36] Jain S, Kamimoto L, Bramley AM, Schmitz AM, Benoit SR, Louie J, Hospitalized patients with 2009 H1N1 influenza in the United States, April–June 2009. N Engl J Med. 2009;361:1935–44. 10.1056/NEJMoa090669519815859

[37] Domínguez-Cherit G, Lapinsky SE, Macias AE, Pinto R, Espinosa-Perez L, de la Torre A, Critically ill patients with 2009 influenza A(H1N1) in Mexico. JAMA. 2009;302:1880–7. 10.1001/jama.2009.153619822626

# Research article: Deaths Associated with Pandemic (H1N1) 2009 among Children, Japan, 2009–2010

**DOI:** 10.3201/eid1711.110649

**Authors:** Akihisa Okumura, Satoshi Nakagawa, Hisashi Kawashima, Takashi Muguruma, Osamu Saito, Jun-ichi Fujimoto, Chiaki Toida, Shuji Kuga, Toshihiro Imamura, Toshiaki Shimizu, Naomi Kondo, Tsuneo Morishima

**Affiliations:** Author affiliations: Juntendo University Faculty of Medicine, Tokyo, Japan (A. Okumura, T. Shimizu);; National Center for Child Health and Development, Tokyo (S. Nakagawa, T. Muguruma, O. Saito, J. Fujimoto, C. Toida, S. Kuga, T. Imamura);; Tokyo Medical University, Tokyo (H. Kawashima);; Gifu University Graduate School of Medicine, Gifu, Japan (N. Kondo);; Okayama University Graduate School of Medicine, Dentistry and Pharmaceutical Sciences, Okayama, Japan (T. Morishima)

## Earning Medscape CME Credit

To obtain credit, you should first read the journal article. After reading the article, you should be able to answer the following, related, multiple-choice questions. To complete the questions (with a minimum 70% passing score) and earn continuing medical education (CME) credit, please go to www.medscape.org/journal/eid. Credit cannot be obtained for tests completed on paper, although you may use the worksheet below to keep a record of your answers. You must be a registered user on Medscape.org. If you are not registered on Medscape.org, please click on the New Users: Free Registration link on the left hand side of the website to register. Only one answer is correct for each question. Once you successfully answer all post-test questions you will be able to view and/or print your certificate. For questions regarding the content of this activity, contact the accredited provider, CME@medscape.net. For technical assistance, contact CME@webmd.net. American Medical Association’s Physician’s Recognition Award (AMA PRA) credits are accepted in the US as evidence of participation in CME activities. For further information on this award, please refer to http://www.ama-assn.org/ama/pub/category/2922.html. The AMA has determined that physicians not licensed in the US who participate in this CME activity are eligible for *AMA PRA Category 1 Credits*™. Through agreements that the AMA has made with agencies in some countries, AMA PRA credit may be acceptable as evidence of participation in CME activities. If you are not licensed in the US, please complete the questions online, print the certificate and present it to your national medical association for review.**Article Title: Deaths Associated with Pandemic (H1N1) 2009 among Children, Japan, 2009–2010**

## Medscape CME Questions

1. You are seeing a 4-year-old girl with a 2-day history of fever, malaise, and cough. Two other children in her day care were diagnosed with pandemic (H1N1) 2009 last week. Her parents are concerned with the possibility of serious outcomes, including death, associated with a potential pandemic (H1N1) 2009 infection.What was the most common symptom among children in the current case series of fatal pandemic (H1N1) 2009 cases?A. CoughB. RhinorrheaC. Altered mental statusD. Fever2. What were the 2 most common causes of death in the current case series of children with pandemic (H1N1) 2009 infection?A. Respiratory failure and viral sepsisB. Encephalopathy and viral sepsisC. Myocarditis and respiratory failureD. Encephalopathy and cardiopulmonary arrest3. What other characteristics of the specific causes of death associated with pandemic (H1N1) 2009 infection should you consider in the evaluation and treatment of this patient?A. 90% of children who died of cardiopulmonary arrest had important preexisting medical conditionsB. Chest radiographs demonstrated bilateral infiltrates among children who died of cardiopulmonary arrestC. All children with encephalopathy had evidence of brain edema on neuroimaging studiesD. Children who died of respiratory failure were generally free of preexisting medical conditions4. Which of the following variables was most significantly related to the cause of death of children in the current study?A. Older age was associated with a higher risk of cardiopulmonary arrestB. Cardiopulmonary arrest occurred close to the onset of illness compared with other causes of deathC. Treatment with neuraminidase inhibitors reduced the overall risk of mortalityD. Respiratory or neurologic disorders were more frequent among children with death due to respiratory failure

## Activity Evaluation

**Table:** 

1. The activity supported the learning objectives.				
Strongly Disagree				Strongly Agree
1	2	3	4	5
2. The material was organized clearly for learning to occur.				
Strongly Disagree				Strongly Agree
1	2	3	4	5
3. The content learned from this activity will impact my practice.				
Strongly Disagree				Strongly Agree
1	2	3	4	5
4. The activity was presented objectively and free of commercial bias.				
Strongly Disagree				Strongly Agree
1	2	3	4	5
